# Is oxygen availability a limiting factor for *in vitro* folliculogenesis?

**DOI:** 10.1371/journal.pone.0192501

**Published:** 2018-02-09

**Authors:** Riccardo Talevi, Sam Sudhakaran, Vincenza Barbato, Anna Merolla, Sabrina Braun, Maddalena Di Nardo, Valentina Costanzo, Raffaele Ferraro, Nicola Iannantuoni, Gerardo Catapano, Roberto Gualtieri

**Affiliations:** 1 Dipartimento di Biologia, Università di Napoli "Federico II", Complesso Universitario di Monte S Angelo, Napoli, Italy; 2 St Bartholomew's Hospital, W Smithfield, London, United Kingdom; 3 Genesis Day Surgery sdc, Caserta, Italy; 4 Ospedale S. Maria delle Grazie, ASL Napoli 2 Nord, Località La Schiana, Pozzuoli, Italy; 5 Department of Environmental and Chemical Engineering, University of Calabria, Rende, Italy; National Cancer Institute, UNITED STATES

## Abstract

Transplantation of ovarian tissue for the preservation of fertility in oncological patients is becoming an accepted clinical practice. However, the risk of re-introducing tumour cells at transplantation has stirred an increased interest for complete in vitro folliculogenesis. This has not yet been achieved in humans possibly for the lack of knowledge on the environmental milieu that orchestrates folliculogenesis *in vivo*. The main aim of this study was to investigate the effect of oxygen availability on follicle health and growth during in vitro culture of ovarian tissue strips. To this end, a model was developed to predict the dissolved oxygen concentration in tissue under varying culture conditions. Ovarian cortical strips of bovine, adopted as an animal model, and human tissue were cultured in conventional (CD) and gas permeable (PD) dishes under different media column heights and gaseous oxygen tensions for 3, 6 and 9 days. Follicle quality, activation of primordial follicles to the primary stage, and progression to the secondary stage were analysed through histology. Follicle viability was assessed through a live-dead assay at the confocal scanning laser microscope. Findings showed a higher follicle quality and viability after culture of bovine ovarian strips in PD in adequate medium height and oxygen tensions. The best culture conditions found in the bovine were adopted for human ovarian strip culture and promoted a higher follicle quality, viability and progression. Overall, data demonstrated that modulation of oxygen availability in tissue plays a key role in maintaining follicles’ health and their ability to survive and progress to the secondary stage during ovarian tissue in vitro culture. Such culture conditions could increase the yield of healthy secondary follicles for subsequent dissection and individual culture to obtain competent oocytes.

## Introduction

Advancements in anti-cancer therapy have improved the survival rates of cancer patients [1] and increased the focus on post-treatment quality of life and their future reproductive potential. Anti-cancer regimes, like chemotherapy and ionizing radiations, induce variable degrees of irreversible premature ovarian failure in 100% of patients, threatening their fertility even after complete cancer remission [2,3]. Ovarian tissue cryopreservation is a promising fertility preservation strategy and represents the only option available for pre-pubertal cancer patients due to their ovarian dormancy at this age [4–7]. Thus far, orthotopic ovarian tissue transplantation following cryopreservation has yielded more than 86 pregnancies worldwide [8] suggesting the future potential of this technique.

However, transplantation of ovarian tissue following cryopreservation is approached with extreme caution, particularly in the case of blood-borne and highly metastatic malignancies, where the possibility of re-introducing tumour cells back into the patient after cancer remission represents a serious risk [9,10]. Lately, the fact that the follicular basal lamina hinders cancer invasion into the oocyte and follicular cells [11] has triggered widespread interest in the development of culture systems for primordial ovarian follicles that constitute the major share of ovarian reserve in women of all age groups. Several strategies for *in vitro* follicular growth have been proposed over the years in which follicles were isolated either at the primordial stage or, after a first culture step of ovarian cortical strips, at the secondary stage, and then cultured until full maturation [12–16].

Ovarian strip *in situ* culture has tremendous advantages over isolated primordial follicle culture systems because follicles are maintained within their natural environment. The ovarian tissue itself is a regulating force, providing cells with an array of highly complex and dynamic bio-mechanical signals [17] which are very difficult to accurately mimic *in vitro* with an artificial matrix. Success of *in vitro* follicular culture in the murine model has confirmed the important role played by initiating primordial follicle growth *in situ* within the ovarian tissue in a two-step culture approach [12].

Unfortunately, the success of current ovarian tissue culture systems in the human is still unsatisfactory being limited by the low efficiency of long-term survival and growth of primordial and primary follicles *in situ* [18,19]. Over the years, several studies have investigated ovarian tissue nutritional and endocrine requirements with the aim to optimise media for cortical strip culture [20,21]. Nonetheless, the yield of secondary follicles seldom exceeded 10% of the total follicle population obtained post-culture [15,22]. It has been proposed that the inefficient transport of oxygen could be a major hurdle in establishing optimal ovarian tissue cultures because it may hinder oocyte/follicle development and cause tissue necrosis [23]. Only a few studies have investigated the importance of oxygen supply for follicular growth, and results are still controversial [23–27].

The main aim of this study was to investigate the influence of oxygen availability to ovarian cells (e.g. the dissolved oxygen concentrations at the surfaces of the ovarian strip, C_O2,TS_) during in vitro culture on quality, viability and progression of human follicles. To save precious human biological material, adequate culture conditions were identified in preliminary experiments performed in the bovine, and were then used for the culture of human ovarian tissue. To this end, C_O2,TS_ was modulated by using conventional dishes (CD) or gas-permeable dishes (PD), by varying media column height above the strips, and by incubation in air or 5% O_2_.

## Materials and methods

### Chemicals and consumables

Lumox gas-permeable culture dishes and conventional culture dishes 50mm in diameter were from Sarstedt (Nümbrecht, Germany) and Falcon (Sigma-Aldrich, Milan, Italy) respectively. Leibovitz’s L-15 medium, α-MEM Glutamax medium (code number 32571), Insulin transferrin selenium (ITS) 100x, Live/dead Fixable far red stain were purchased from Invitrogen (Milan, Italy). Penicillin streptomycin 100x, Amphotericin B 250μg/ml, Bovine serum albumin (BSA), L-Ascorbic acid, L-Glutamine 200mM, Hoechst 33342, Fructose, α-thioglycerol and Eosin-Y were purchased from Sigma Aldrich (Milan, Italy). Mayers’s hematoxylin and paraffin wax were from Carlo Erba (Milan, Italy).

### Collection and preparation of ovarian tissue

Bovine ovaries were collected at the Slaughterhouse Straccione (San Marcellino, Caserta, Italy; CEE accreditation number 1403/M) and transported to the laboratory in Leibovitz’s L-15, 1% penicillin-streptomycin (Pen-Strep), 1μg/ml Amphotericin-B, at 4°C, within 2h of slaughter.

The use of human tissue was approved by the Ethics Committee of Regione Campania (ASL NA1 Centro, Naples, Italy; reference number 57 CE 2–2017). After obtaining written informed consent, ovarian biopsies were collected from six women (age = 27.1 ± 6.9 years; range 18–34 years) during laparoscopic surgery for benign gynecologic conditions and transported to the lab as described above.

Bovine ovaries and human ovarian biopsies were transferred to handling medium (Leibovitz’s L-15, 2mM glutamine, 3mg/ml BSA, 1% Pen-Strep, 1μg/ml amphotericin B) and cortical slices (~ 0.5mm thick) were manually dissected at room temperature (RT) avoiding areas with visible antral follicles to ensure a predominant primordial follicle population. The cortical slices were further uniformly sliced into 1mm x 1mmx 0.5mm strips using a tissue chopper (Mcilwain, Mickle Laboratory Engineering Company, Ltd, Surrey, UK). The strips were pooled in a 10cm Petri dish, mixed by gentle agitation, washed twice in fresh handling medium, and 10 strips were randomly distributed into each culture dish. Fresh control strips from each ovary were processed for histology and viability assessment as a control.

### Strip culture and modulation of oxygen availability

In each experiment, cortical strips from the same ovary were cultured in α-MEM, 3mM glutamine, 0.1% BSA, 1% Pen-Strep, 1% ITS (10 μg/ml Insulin, 5.5 μg/ml Transferrin, 6.7 ng/ml Selenium), 1μg/ml amphotericin-B, 50μg/ml ascorbic acid at 37°C, 5% CO_2_ and 95% humidity in air. Half medium was changed every 48h. At the end of culture, 5 strips from each dish were treated for histology and 5 strips for viability assessments.

To identify the culture conditions ensuring the most adequate oxygen availability (e.g. dissolved oxygen concentrations at the upper, C_O2,TSU_, and bottom surface, C_O2,TSB_, of the ovarian strip), oxygen supply was modulated by culturing ovarian strips in conventional (CD) or gas permeable dishes (PD) in a volume of medium yielding average column heights from the dish bottom of 1.4mm (HV: high volume, 5ml) or 0.7mm (LV: low volume, 2.5ml) in 5% CO_2_ in air or in 5% CO_2_, 5% O_2_, 90% N_2_. The most adequate culture conditions found in the bovine model were applied to human ovarian tissue culture.

### Experimental design

In experiment I (n = 3), bovine ovarian strips (BOSs) from the same ovary were cultured as described above in 5ml of medium in permeable vs conventional dishes (conditions hereinafter referred to as PDHV and CDHV, respectively) for 3, 6 and 9 days in 5% CO_2_ in air.

Results of experiment I demonstrated a significantly higher follicle quality and viability in PD versus CD both at day 6 (D6) and 9 (D9). Hence, in experiment II (n = 3) BOSs from the same ovary were cultured in 5% CO_2_ in air for 6 days in high or low volumes of medium in PD vs CD (conditions hereinafter referred to as PDHV, PDLV, CDHV, CDLV, respectively).

In PD, the strip bottom surface is in direct contact with a gas-permeable polymer which ensures direct oxygen supply and also metabolic CO_2_ removal across such surface. Experiment III (n = 3) was designed to investigate whether the better outcome of BOSs cultured in PDHV in experiment I and II was caused by an adequate oxygen availability or an enhanced removal of metabolic CO_2_. To this end, BOSs were cultured in CDHV and PDHV for 6 days under 5% CO_2_ in air vs 5% CO_2_, 5% O_2_, 90% N_2_.

Experiment IV (n = 6) was aimed to investigate whether the most adequate culture condition found in bovine experiments could have similar effects on human follicle quality, activation, progression and viability. To this end, human ovarian strips (HOSs) from the same ovaries were cultured in PDHV vs CDHV under 5% CO_2_ in air for 6 and 9 days.

### Model of oxygen transport in ovarian tissue

**Fig 1:**
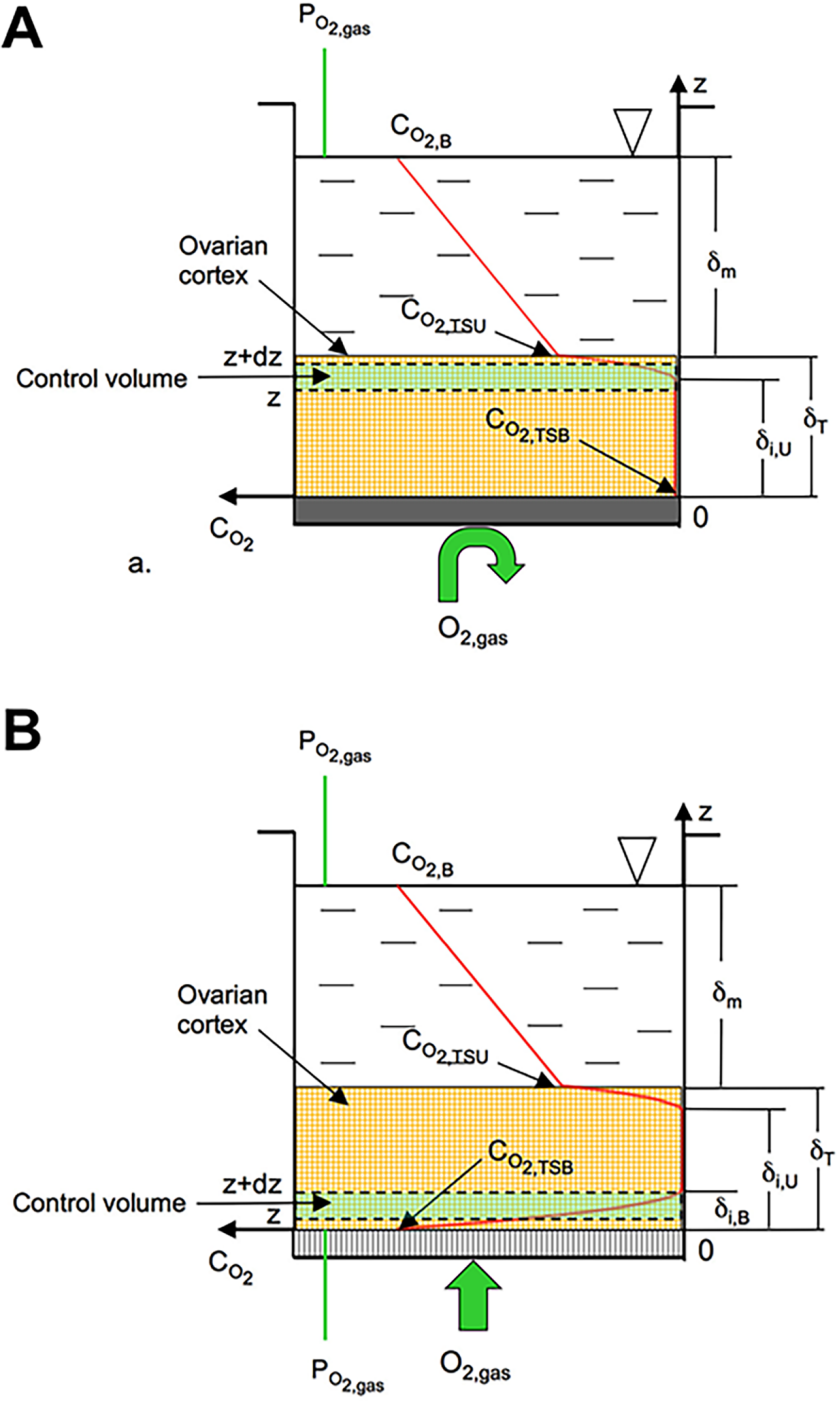
Scheme of ovarian cortex culture in conventional (CD) and gas-permeable (PD) dishes. The continuous red lines show exemplary dissolved oxygen profiles predicted in medium and tissue by the oxygen transport model described in Eqs 1–3 when strips of ovarian cortical tissue are cultured in: A) conventional dishes with gas-impermeable bottom (CD); B) dishes with gas-permeable bottom (PD). The green box identifies the control volume with respect to which the differential mass balance equations were written, as described in S1 Appendix and S2 Appendix for CD and PD, respectively. The meaning of the symbols may be found in text.

Dissolved oxygen concentration in ovarian cortical tissue during *in vitro* culture is generally much lower than that in air because of the poor solubility of oxygen in medium, and of the resistance to oxygen diffusion in medium and tissue combined with oxygen consumption by ovarian cells (Fig 1). To account for such effects and gather more realistic information than those derived from the incubator setting, dissolved oxygen concentration anywhere in tissue (C_O2,T_) under varying culture conditions was predicted with a model describing oxygen diffusive transport in medium and tissue at steady state. It was assumed that tissue has uniform properties, that oxygen enters tissue only across the upper surface (i.e. CD) or also from the bottom strip surface (i.e. PD), and that an anoxic zone forms amid the strip thickness. In CD, it is C_O2,T_ >0 only in the uppermost strip part (i.e. from z>δ_i,U_ to z = δ_T_), it decreases towards the strip bottom and levels off to zero at a fractional distance δ_i,U_/ δ_T_ from it (i.e. C_O2,T_ = 0 from z = 0 to z = δ_i,U_). In PD, C_O2,T_ at the strip bottom is assumed equal to the dissolved oxygen concentration in medium equilibrating the gaseous oxygen tension (C_O2,B_). As an effect, the dissolved oxygen concentration is C_O2,T_>0 also near the strip bottom but it decreases towards the upper strip surface and levels off to zero at a fractional distance δ_i,B_/δ_T_ from the bottom (i.e. C_O2,T_ = 0 from z = δ_i.B_ to z = δ_i,U_). The bias associated to the lack of reliable estimates of ovarian cortex structural, transport and metabolic properties was minimized by lumping such properties in the dimensionless Thiele modulus ϕ and mass Biot number Bi_m_, actually determining the dissolved oxygen concentration profile in tissue. The former compares the rate of oxygen metabolic consumption to diffusion in tissue. The latter compares the dissolved oxygen concentration drop in tissue to that in medium to sustain oxygen transport to cells. In CD, an oxygen mass balance about a tissue control volume infinitesimal in z yields the dimensionless dissolved oxygen concentration, C_O2,T_ /C_O2,B_, in the uppermost strip part at a fractional distance z/ δ_T_ from the bottom as follows (S1 Appendix):

$$\frac{C_{O2,T}(z/\delta_{T})}{C_{O2,B}}=\frac{1}{2}\phi^{2}[{\left(\frac{\delta_{i}}{\delta_{T}}\right)}^{2}+{\left(\frac{z}{\delta_{T}}\right)}^{2}-2\frac{\delta_{i}}{\delta_{T}}\frac{z}{\delta_{T}}]$$ (1)

with

$$\begin{array}{l} \frac{\delta_{i}}{\delta_{T}}=1-[\frac{1}{\phi }\sqrt{\frac{\phi^{2}}{Bi_{m}}+2}-\frac{1}{Bi_{m}}];\;\phi^{2}=\frac{G\prime \prime \prime }{D_{T}C_{O2,B}}\delta_{T}^{2};\;Bi_{m}=\frac{D_{m}\delta_{T}}{\delta_{m}D_{T}} \end{array}$$ (2)

where: G^’’’^ is thse volumetric tissue oxygen consumption rate; *D*_T_ and *D*_m_ are oxygen diffusivity in tissue and medium, respectively; δ_m_ and δ_T_ are the medium height above the strip and tissue thickness, respectively. In PD, C_O2,T_/ C_O2,B_ close to the strip upper surface is the same as in Eqs 1 and 2. C_O2,T_/ C_O2,B_ close to the strip bottom may be gathered with a similar mass balance by imposing that C_O2,T_ equals C_O2,B_ at z = 0 and that it levels off to zero at z = δ_i,B_, to give (S2 Appendix):

$$\frac{C_{O2,T}(z/\delta_{T})}{C_{O2,B}}=[1+\frac{1}{2}\phi^{2}{\left(\frac{z}{\delta_{T}}\right)}^{2}-\sqrt{2}\;\phi (\frac{z}{\delta_{T}})].$$ (3)

To gather qualitative information on oxygen availability in the ovarian strips for the conditions used for this work, exemplary profiles of the dimensionless dissolved oxygen concentration in tissue, C_O2,T_/C_O2,B_, were estimated from Eqs 1–3 for representative parameter values, as follows: G^’’’^ = 2x10^-2^ mol/(s m^3^); *D*_m_ = 3.5x10^-9^ m^2^/s; *D*_T_ = 2.8x10^-9^ m^2^/s; δ_T_ = 0.4–0.5 mm; δ_m_ = 0.2 (LV) or 0.9 (HV) mm. C_O2,B_ was set at 0.2 mol/m^3^ (i.e. pCO2_gas_ = 21%), unless otherwise noted. These values yield the following dimensionless parameters values: ϕ = 2.5, Bi_m_ = 0.7 (HV) or 3 (LV). Oxygen availability to ovarian cells was characterized in terms of the dissolved oxygen concentrations at the upper (C_O2,TSU_) and bottom (C_O2,TSB_) strip surface, the average dissolved oxygen concentration in the strip (C_O2,T,avg_), and the percent strip volume operated under anoxic conditions.

### Histology

To assess follicular quality, activation and progression, strips were fixed in Bouin’s, dehydrated in increasing ethanol concentrations, embedded in paraffin and 5μm serial sections were stained with hematoxylin and eosin. All follicles visualized in serial sections from each strip were graded and staged by two blinded expert observers. Follicles were evaluated only when the germinal vesicle was visible to minimize the chance of re-counting. Follicle quality was graded as previously reported [28]. Briefly: grade 1 follicles were spherical and had homogeneously distributed granulosa cells (GCs) and an oocyte with homogenous cytoplasm and slightly granular nucleus, in the center of which condensed chromatin in the form of a dense spherical structure is detected; grade 2 follicles had GCs pulled away from the edge of the follicle but still a spherical oocyte; and grade 3 follicles had GCs with pyknotic nuclei and misshapen oocyte with or without vacuolation. Follicle stages were scored according to Gougeon's criteria [29], as follows: primordial with a single layer of flat GCs; primary with a complete single layer of cuboidal GCs; secondary with two or more complete layers of cuboidal GCs.

### Viability assessment

Strips were incubated in Dulbecco’s PBS with 1μg/ml Live/Dead Fixable Far Red Stain and 10μg/ml Hoechst 33342, 3 hours at 4°C under gentle agitation, fixed in 4% paraformaldehyde in PBS 2 hours at RT, washed in fresh PBS and incubated in PBS 10μg/ml Hoechst 33342 at 4°C overnight [30]. The live/dead probe is resistant to fixation, reacts with free amines both in the cell interior and on the cell surface and is excluded by cells with intact membranes. Strips were then optically cleared using See DB clearing protocol [31]. Briefly, samples were serially incubated in 5 mL of 20%, 40%, and 60% (wt/vol) fructose, each for 3 hours, 80% and 100% fructose (wt/vol) each for 12 hours, and finally in 115% (wt/vol) fructose for 24 hours with gentle shaking at RT. All fructose solutions were supplemented with 0.5%. α-thioglycerol. To avoid compression, strips were mounted in 115% fructose on a glass slide with 3 spacer coverslips (0.17mm) placed on each side and covered with a coverslip.

Analysis was carried out with a Leica TCS SP5 confocal scanning laser microscope (Leica Microsystems, Wetzlar, Germany) using a 405-nm diode laser for visualizing the nuclear label (Hoechst 33342) and a 633-nm helium neon laser for the live/dead probe. Each strip was traversed using the z-position control and fields to a depth of 300μm from the tissue surface were imaged using a 63x glycerol immersion objective.

### Statistical analysis

For each experiment, data is presented as cumulative percentages. Overall, statistical analysis was performed by Fisher’s exact test for pairwise comparisons when overall significance was detected.

## Results

### Model-predicted oxygen availability

As shown in Table 1, when strips were cultured in air, oxygen availability increases with the culture conditions in the following order: CDHV<CDLV<PDHV<PDLV. When strips were cultured at pCO2_gas_ = 5% in CDHV the model predicts that 99% of the strip volume is operated under anoxic conditions. Use of dishes with a gas-permeable bottom in PDHV slightly enhances oxygen availability, yet more than 85% of the strip volume is operated under anoxic conditions.

**Table 1:** Model-predicted oxygen availability in a tissue strip.

Culture condition	C_O2,TSB_/C_O2,B_, %	C_O2,TSU_/C_O2,B_, %	C_O2,T,avg_/C_O2,B_, %	Anoxic strip volume fraction, %
CDHV	0	3.6	0.053	89.3
PDHV	100	3.6	1.25	32.6
CDLV	0	32.6	0.3	67.7
PDLV	100	32.6	1.55	11.1

Model predictions were obtained for ϕ = 2.5 and Bi_m_ = 0.7 (HV) and 3 (LV): C_O2,TSB_−dissolved oxygen concentration at strip bottom surface; C_O2,TSU_−dissolved oxygen concentration at strip upper surface; C_O2,T,avg_−average dissolved oxygen concentration in the strip volume.

### Experiment I

**Fig 2:**
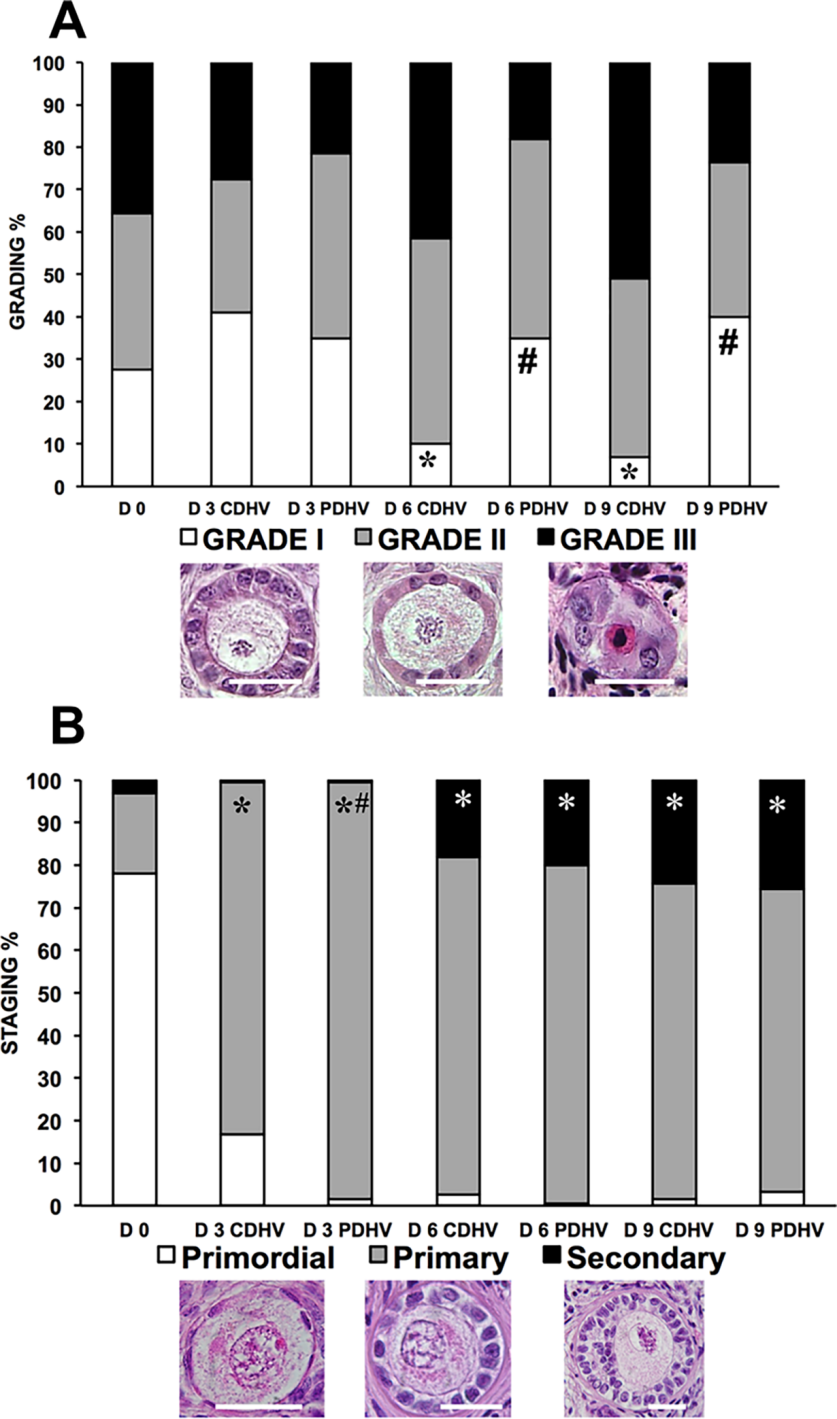
Grading and staging of bovine follicle in strips cultured in air in PDHV vs CDHV. Histological grading (A) and staging (B) of bovine follicles in strips cultured in PDHV and CDHV in air. Bar = 20μm. ^*^P<0.01 vs D0; ^#^P<0.01 vs corresponding treatments.

Histological analysis (n = 1837 follicles: D0, 307; D3 CDHV, 242; D3 PDHV, 265; D6 CDHV, 287; D6 PDHV, 223; D9 CDHV, 275; D9 PDHV, 238) showed that strips cultured in PDHV harboured significantly more grade I follicles both on D6 and 9. By D6 the proportion of grade I follicles in CDHV dropped significantly compared to day 0 (Fig 2A). Both groups (Fig 2B) showed a marked and significant decrease of primordial follicles and a corresponding significant increase of primary follicles at D3 as compared to D0. Interestingly, both groups showed a significantly higher proportion of secondary follicles at D6 and D9 than at D0.

**Fig 3:**
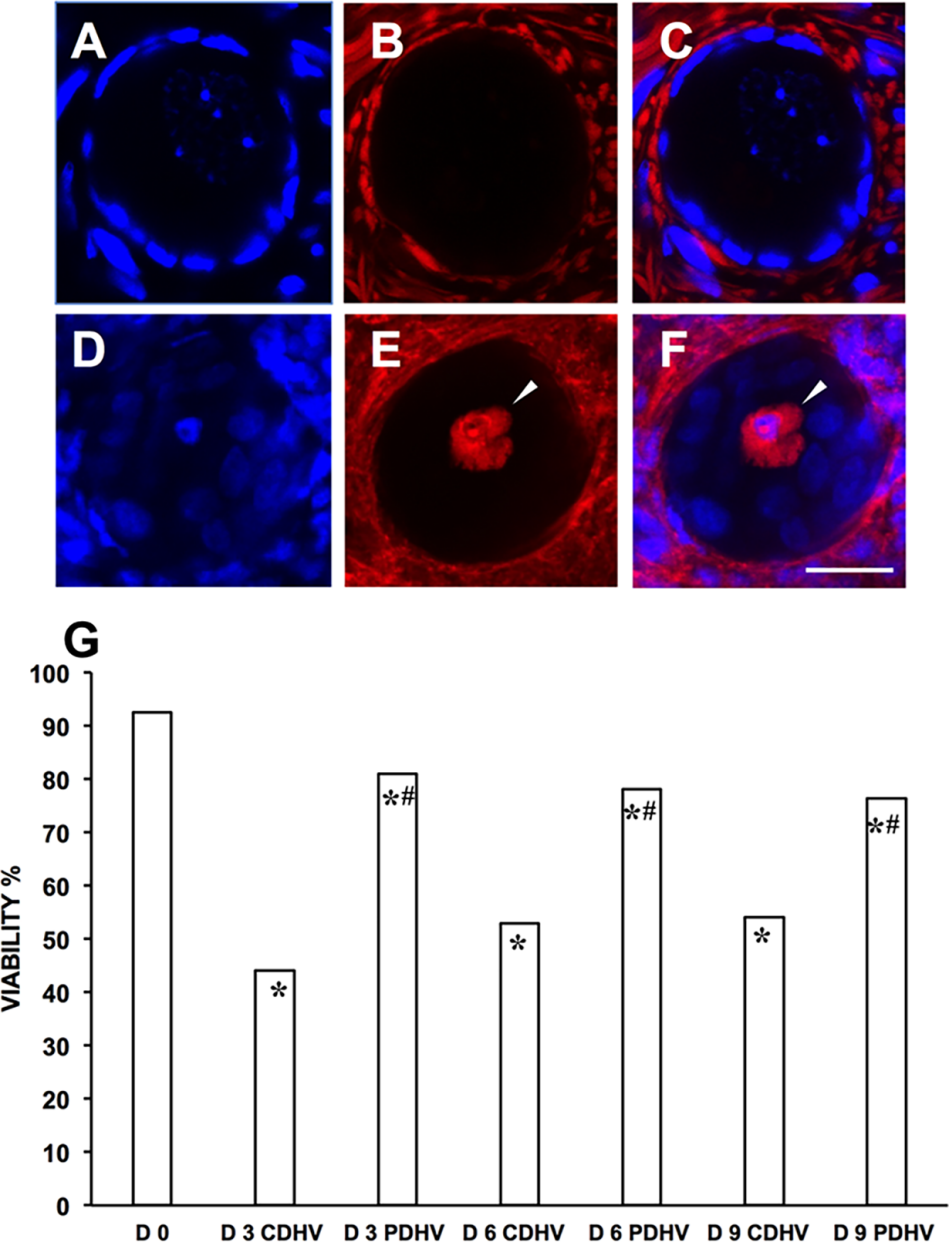
Viability of bovine follicles in strips cultured in air in PDHV vs CDHV. Representative confocal micrographs of a live (A,B,C) and a dead (D,E,F) follicle. (A,D) Hoechst 33342–stained nuclei; (B,E) live–dead far-red probe; (C,F) merge. (G) Follicle viability in fresh and cultured strips. Arrowheads indicate a dead oocyte. Bar = 20μm. ^*^P<0.01 vs d0; ^#^P<0.01 vs corresponding treatments.

Follicle viability was obtained from a total of 1969 follicles (number of follicles: D0, 254; D3 CDHV, 278; D3 PDHV, 312; D6 CDHV, 323; D6 PDHV, 236; D9 CDHV, 202; D9 PDHV, 364). Fig 3 shows representative confocal micrographs of one viable (A-C) and one dead follicle (D-F). Viability significantly decreased during culture under both conditions. However, viability throughout the culture was about 80% in PDHV and only around 50% in CDHV (Fig 3G).

Taken together, this data (S1 Table) suggests that increased C_O2,TS_ enhances follicular activation and preserves viability.

### Experiment II

**Fig 4:**
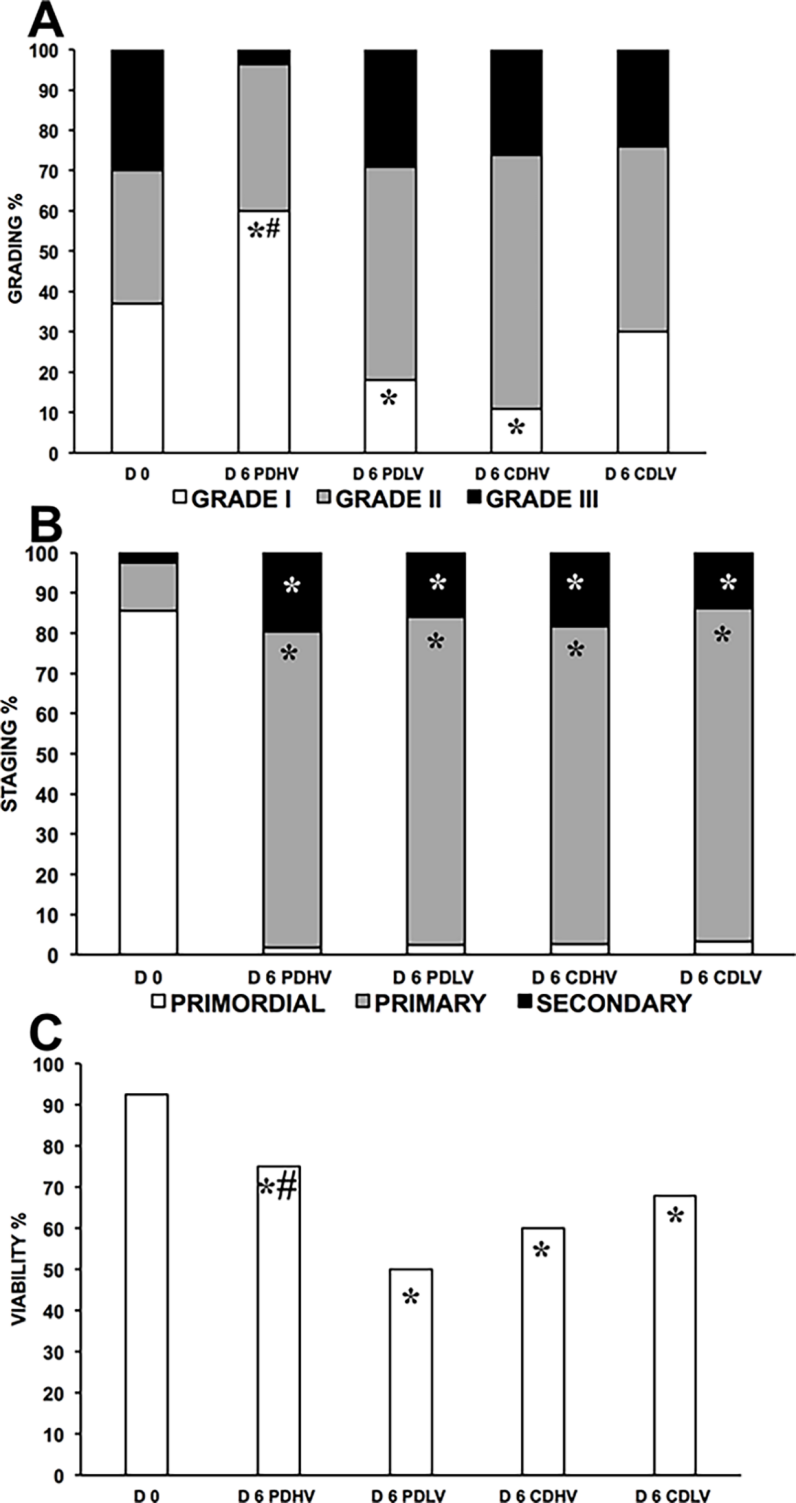
Quality of bovine follicles in strips cultured under various conditions in air. Histological grading (A), staging (B), and viability (C) of bovine follicles in strips cultured in PDHV, PDLV, CDHV and CDLV in air. *P<0.01 vs D0; #P<0.01 vs corresponding treatments.

The transport model predicts that the combined use of different medium heights and CD or PD yielded progressively increasing oxygen availability in the following order: CDHV<CDLV<PDHV<PDLV (Fig 1 and Table 1). Histological analysis (n = 1243 follicles: D0, 273; D6 PDHV, 213; D6 PDLV, 287; D6 CDHV, 199; D6 CDLV, 271) showed that culture in PDHV yielded a significantly higher percentage of grade 1 follicles than both D0 and the other D6 samples (Fig 4A, S2 Table). The higher grading in PDHV compared to the fresh control could be due to the re-absorption of atretic follicles during culture coupled with no further addition of atretic follicles. Grading under the four conditions reflects the model-predicted oxygen availability in tissue. In fact, culture in CDHV and PDLV at the predicted lowest and highest oxygen availability yielded the worst follicle quality. Culture in CDLV outperformed CDHV in terms of grade I follicles possibly showing a positive effect of higher C_O2,T_ caused by reduced media height.

By D6, the majority of the primordial follicles was activated to the primary stage and a significant progression to the secondary stage was observed compared to D0 with no significant differences among groups (Fig 4B, S2 Table).

Viability (n = 971 follicles: D0, 237; D6 PDHV, 173; D6 PDLV, 187; D6 CDHV, 148; D6 CDLV, 226) was 89.5% at D0 and significantly dropped in all groups at D6 (Fig 4C, S2 Table). Viability at D6 in PDHV was significantly better than in PDLV and CDHV, whereas in CDLV it was only slightly lower than in PDHV.

### Experiment III

**Fig 5:**
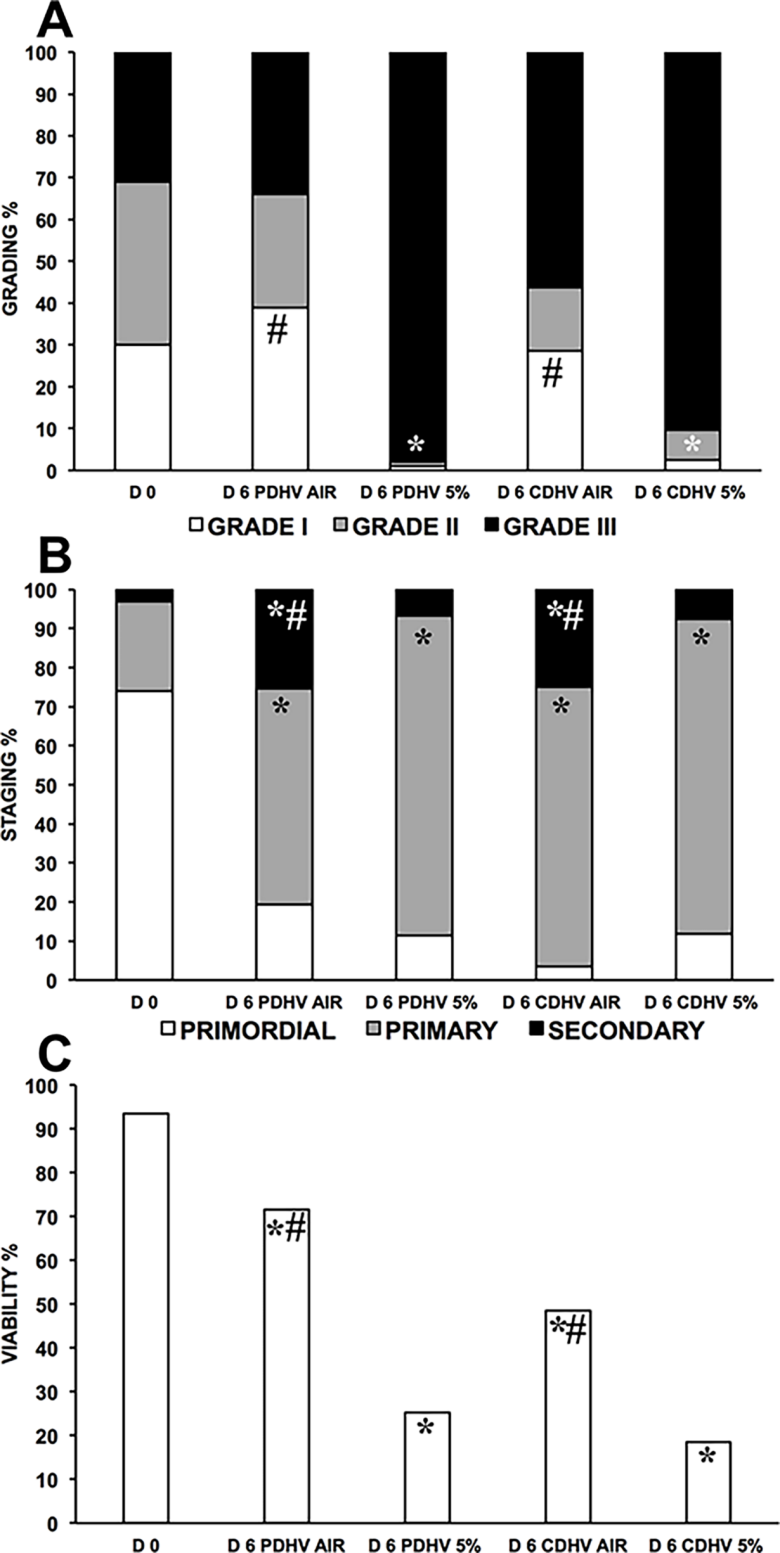
Quality of bovine follicles in strips cultured in air vs. 5%O2. Histological grading (A), staging (B), and viability (C) of bovine follicles in strips cultured in PDHV and CDHV in air versus 5% O2. ^*^P<0.01 vs D0; ^#^P<0.01 vs corresponding treatments.

Histological analysis (n = 978 follicles: D0, 222; D6 PDHV AIR, 187; D6PDHV 5%, 148; D6 CDHV AIR, 265; D6 CDHV 5%, 156) showed that follicle quality was best in PDHV in air. In agreement with the large anoxic strip fraction predicted by the model, culture in 5% O_2_ markedly and significantly reduced follicle quality in both CDHV and PDHV (Fig 5A, S3 Table). All conditions supported high follicle activation, but culture under 5% O_2_ impaired progression to the secondary stage (Fig 5B, S3 Table). Viability analysis (n = 587 follicles: D0, 143; D6 PDHV AIR, 96; D6PDHV 5%, 109; D6 CDHV AIR, 121; D6 CDHV 5%, 118) indicates that PDHV in air provides the best culture conditions (Fig 5C, S3 Table).

### Experiment IV

**Fig 6:**
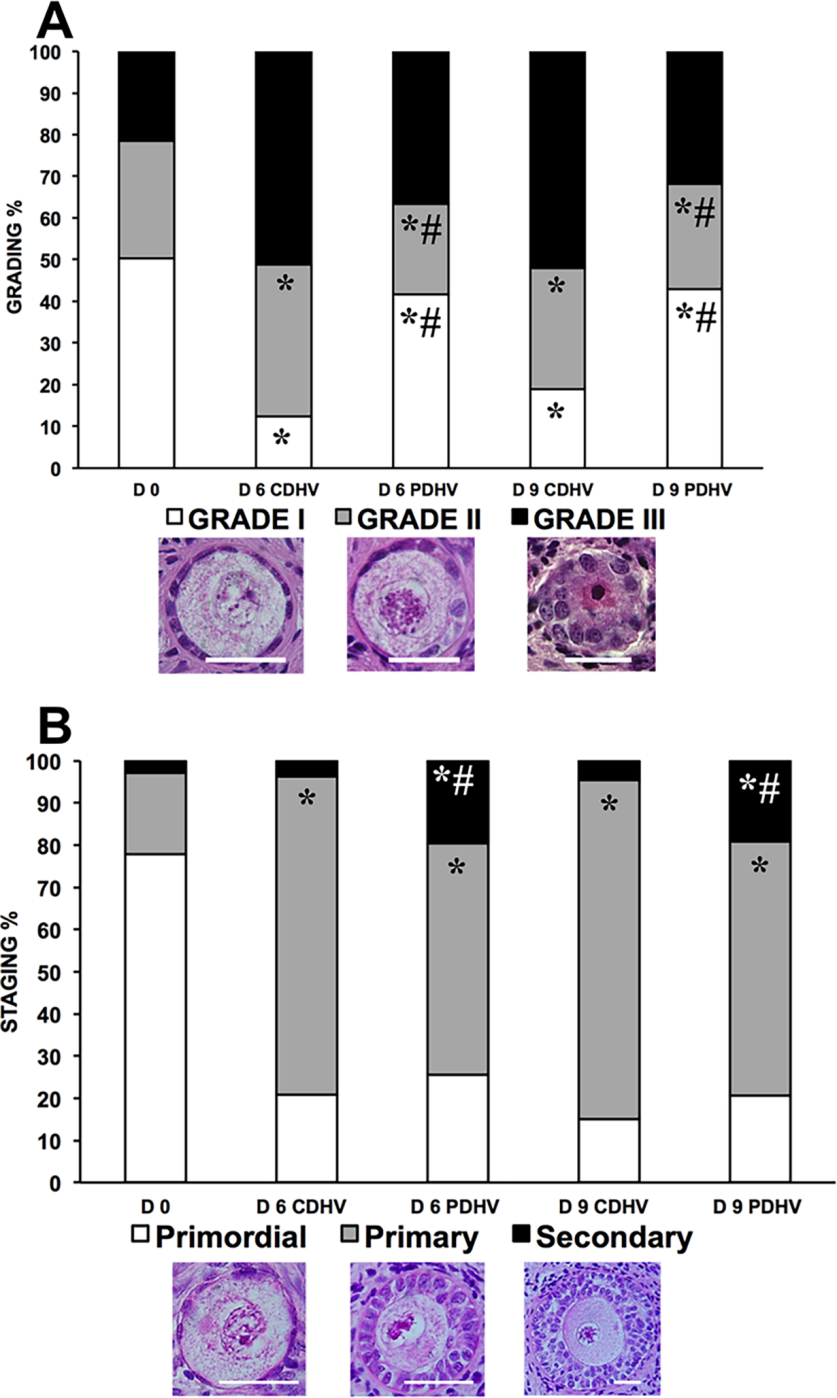
Grading and staging of human follicles in strips cultured in air in PDHV vs CDHV. Histological grading (A) and staging (B) of human follicles in strips cultured in PDHV and CDHV. Bar = 20μm. ^*^P<0.01 vs D0; ^#^P<0.01 vs corresponding treatments.

**Fig 7:**
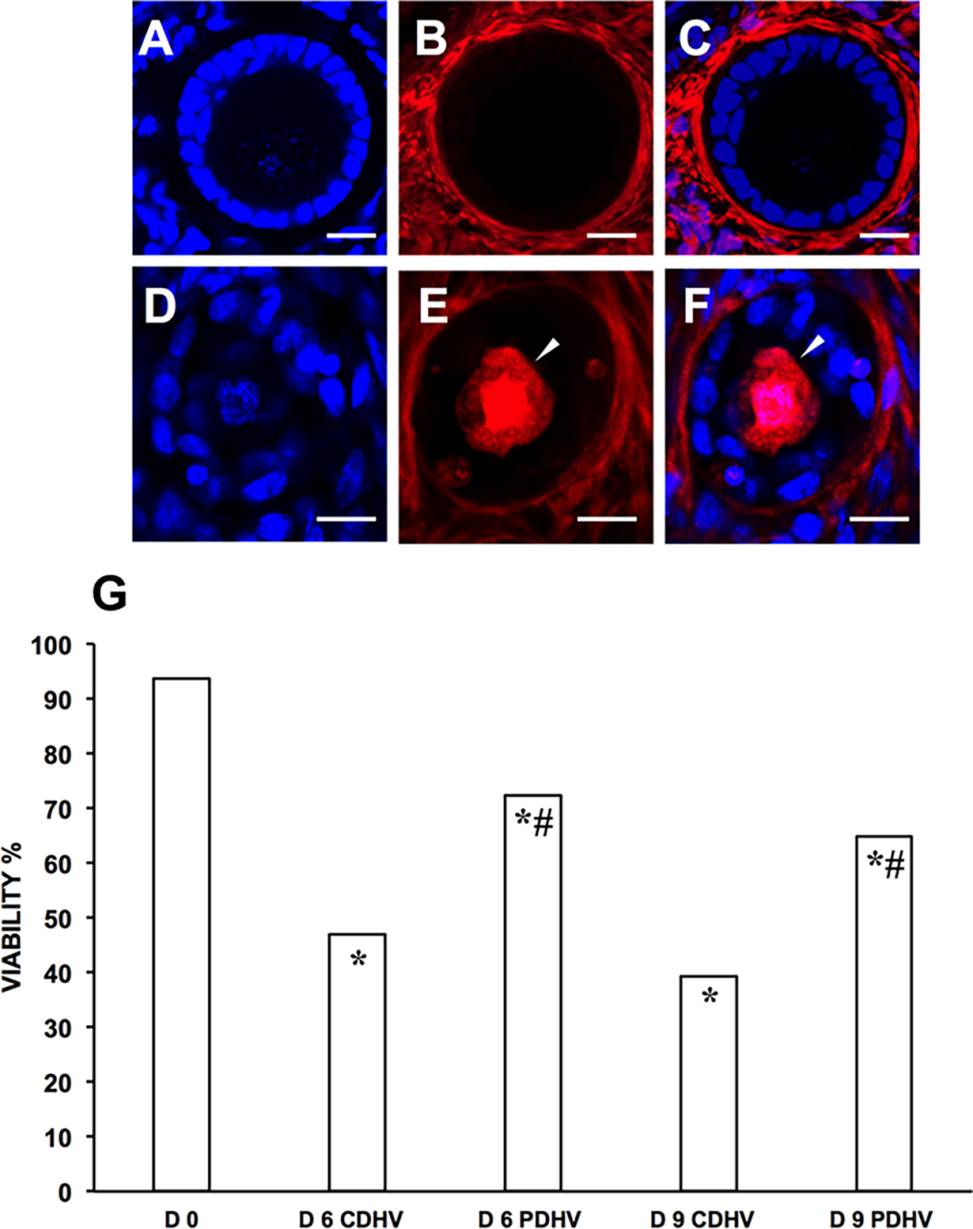
Viability of human follicles in strips cultured in PDHV vs CDHV. Representative confocal micrographs of a live (A,B,C) and a dead (D,E,F) follicle. (A,D) Hoechst 33342–stained nuclei; (B,E) live–dead far-red probe; (C,F) merge. (G) Follicle viability in fresh and cultured strips. Arrowheads indicate a dead oocyte. Bar = 15μm. *P<0.01 vs D0; ^#^P<0.01 vs corresponding treatments.

Histological analysis of HOSs (n = 1383 follicles: D0, 243; D6 CDHV, 327; D6 PDHV, 302; D9 CDHV, 206; D9 PDHV, 305) (Fig 6A, S4 Table) showed that follicle quality significantly dropped during culture as compared to D0 (Fig 6A). As seen in the bovine, culture in PDHV still provided the best conditions and yielded a greater proportion of grade 1 follicles than in CDHV at both D6 and 9 (Fig 6A). Culture under all conditions supported follicle activation but progression to the secondary stage was significantly higher in PDHV than in CDHV at both D6 and D9 (Fig 6B, S3 Table). In agreement with the histology findings, viability (n = 745 follicles: D0, 133; D6 CDHV, 125; D6 PDHV, 231; D9 CDHV, 119; D9 PDHV, 137) decreased during culture as compared to D0 (Fig 7A–7F). Culture in PDHV provided the best conditions yielding a significantly higher viability than in CDHV both at D6 and D9 (Fig 7G, S4 Table).

## Discussion

Human folliculogenesis in vivo is a complex process that stretches over a period of more than 290 days [32]. Although follicle growth in vitro is exceptionally accelerated [33], to achieve complete in vitro folliculogenesis it is necessary to develop culture strategies and systems that support long-term maintenance of tissue viability and follicle quality. The two-step culture strategy [12] is among the most promising techniques to realise complete in vitro folliculogenesis. Within this framework, the optimisation of cortical strip culture may play a pivotal role in the preservation of follicle health and in its activation and progression to the secondary stage. When ovarian tissue fragments are cultured in conventional culture dishes, it has been proposed that the poor availability of oxygen may hinder oocyte/follicle development and even cause tissue necrosis [23]. Few studies have investigated the importance of oxygen supply for follicular viability and growth but have not provided yet conclusive evidence [23–27]. In such studies, ovarian strips varying in geometry and thickness have been generally cultured at 100% vs 21% or 21% vs 5% pO2_gas_ for various times. Tissue has also been cultured under uncontrolled heights of media differing for composition and supplements.

Albeit simple, the model proposed herein suggests that, in addition to pO2_gas_, the thickness of the ovarian strip, the metabolic rate at which oxygen is consumed (which changes with the tissue source and the culture medium), and the height of medium above the strip, all affect the resistance to oxygen transport from the gas phase, oxygen concentration in the strip, and ultimately oxygen availability to ovarian cells [34]. This suggests that the findings from the reported studies might be contradictory for the multitude of factors that were varied by different investigators and that could affect the actual oxygen availability to ovarian cells. To the best of our knowledge, this is the first time that the importance of oxygen for cortical ovarian tissue is investigated in culture experiments by systematically varying oxygen availability to ovarian cells. The oxygen transport model was instrumental to seeking those conditions that would permit ovarian cells culture in the strip at varying oxygen availability.

Our experiments investigated follicle quality, progression and viability in BOSs and HOSs cultured at varying oxygen availability by using CD or PD, or by changing medium height or gaseous oxygen tension. The overall results indicate that an adequate oxygen supply is required to maintain follicle quality and viability and to promote follicle progression both in bovine and human tissue. The significantly higher proportion of healthy and viable follicles in BOSs cultured in PDHV vs CDHV suggests that culture in CDHV under the conditions used established very low C_O2,T_‘s that compromise follicle health. Culture in PDHV yielded better oxygen availability and preserved follicle quality and viability. Culture under low medium height was used to increase C_O2,T_ in BOSs and mimic the thin medium layer frequently used for strip culture on tissue inserts [34]. Culture in PDHV still offered the best conditions. Culture in CDHV and PDLV, that were predicted to establish the lowest and highest C_O2,T_ respectively, impaired follicle health suggesting that bovine follicles require C_O2,T_ within an optimal range for long-term in vitro culture.

Culture in PDs was expected to enhance oxygen supply by providing oxygen also through the tissue bottom (high C_O2,TBS_), but would also enhance removal of metabolic CO_2_. When BOSs were cultured in PDHV and CDHV in air vs. 5% O_2_, culture under reduced oxygen tension appeared to exert a dominant detrimental effect on follicle quality, viability and progression. This suggests that enhancing CO_2_ removal had less relevant effects on follicle health than enhancing the oxygen availability. This is in agreement with the increased growth, estradiol secretion, and decreased lactate production reported when oxygen availability to mouse preantral follicles was enhanced by culturing them at the medium/air interphase in inverted multiwells plates rather than in immersed upright condition [35]. Our findings are in disagreement with those of Jorssen et al. [24] that reported similar rates of follicle survival and progression in BOS cultured in 5% and 20% oxygen. Such a discrepancy is possibly due to the thinner strips used (0.2 vs 0.5mm in the present study) that may have not limited oxygen diffusion. It should be noted that the goodness of the model predictions is limited by the model assumptions and by the fact that model parameters had to be estimated from literature information for similar tissue under similar conditions to those used in this work for the lack of data. For the sake of the example, *D*_T_ was assumed equal to that reported for ocular stroma [36] because it was thought that it could better account for the heterogeneous nature of the ovarian cortex. The value of G^”‘^ was extrapolated to 6 days of culture from the values reported in the only paper in which the oxygen consumption rate of ovarian cortical strips has been characterized [37].

The best and worst culture conditions identified for bovine tissue (i.e. PDHV and CDHV) were chosen to culture HOSs from six patients. A large number of follicles was analysed for histology and viability. Also for HOSs, culture in PDHV yielded better follicle quality and viability. Different from BOSs, culture in PDHV yielded also a better follicle progression in HOSs. The different culture outcome in the two species could possibly be due to variations in tissue anatomy, metabolism and resistance to oxygen diffusion, and underlies that, in spite of the fact that bovine tissue is considered a reliable model for human in vitro folliculogenesis [38], it may behave differently from human tissue at least in some respects.

Several studies have reported that supplementation of factors like FBS, FSH or activin was not particularly effective in augmenting the growth of primary follicles to the secondary stages [39–42]. It is worth mentioning that in PDHV we obtained one of the highest secondary follicles yield reported in literature by using basal serum-free medium without FSH or activin [43–46]. This suggests that adequate oxygen availability is a key factor to support follicle progression in HOSs culture.

Follicular oxygen requirement is a highly dynamic process in which follicle oxygen consumption increases as follicles progress from the primordial to the primary and secondary stage, outlining the role of both tricarboxylic acid cycle and oxidative phosphorylation in primordial follicle activation and progression [47]. Our results show that all culture conditions promoted follicle activation independent of the predicted oxygen concentration at the strip surfaces or interior, whereas progression to the secondary stage depended more strongly on oxygen availability. This agrees well with the increasingly higher oxygen depletion in tissue caused by the increasing oxygen consumption rates of human follicles as they progress from primordial to primary (2.5-fold) and from primary to the secondary stage (further 3.8-fold) reported by Ishikawa et al. [47].

In conclusion, culture of ovarian strips using gas-permeable dishes under optimal medium height enabled to maintain follicles health and promote their development. We believe that such an approach holds promise to increase the efficiency of a two-step culture strategy by improving quality and yield of secondary follicles for further isolated culture to achieve complete *in vitro* folliculogenesis.

## Supporting information

S1 Appendix Supplementary information on the oxygen transport model in conventional dishes. Details on how the concentration profile in ovarian tissue was obtained when ovarian tissue is cultured in conventional dishes.
(DOCX)

S2 Appendix Supplementary information on the oxygen transport imodel in gas-permeable dishes. Details on how the concentration profile in ovarian tissue was obtained when ovarian tissue is cultured in gas-permeable dishes.
(DOCX)

S1 Table Experiment I. Percentages of bovine follicle grading, staging and viability. H = Histology; V = Viability. Number of follicles analysed are indicated in brackets.
(DOCX)

S2 Table Experiment II. Percentages of bovine follicle grading, staging and viability. H = Histology; V = Viability. Number of follicles analysed are indicated in brackets.
(DOCX)

S3 Table Experiment III. Percentages of bovine follicle grading, staging and viability. H = Histology; V = Viability. Number of follicles analysed are indicated in brackets.
(DOCX)

S4 Table Experiment IV. Percentages of human follicle grading, staging and viability. H = Histology; V = Viability. Number of follicles analysed are indicated in brackets.
(DOCX)
