# Supplementary material for: Is oxygen availability a limiting factor for in vitro folliculogenesis?
Source: PLoS One. 2018 Feb 9;13(2):e0192501. doi: 10.1371/journal.pone.0192501 (PMC5806880; doi:10.1371/journal.pone.0192501)
Supplement: S1 Appendix — Details on how the concentration profile in ovarian tissue was obtained when ovarian tissue is cultured in conventional dishes. (DOCX) [file pone.0192501.s001.docx]

# S1 Appendix

# The dissolved oxygen profile in the upper part of a strip of ovarian tissue cultured in a conventional dish (CD) may be obtained from a steady-state mass balance on oxygen written about the control volume A dz (of infinitesimal thickness dz and surface area A equal to the strip area) in tissue near the upper strip surface as shown in Fig 1, as follows:

# $D_{T} \frac{d^{2}C_{O2,T}\left(z\right)}{dz^{2}} = G'''$ (4)

# subject to the following boundary conditions:

# BC1 z= δ_T_ C_O2,T_=C_O2,TSU_=C_T_ (4a)

# BC2 z=δ_i,U_ dC_O2,T_/dz=0 (4b).

# Integration of Equation 4 yields:

# $C_{O2,T}\left(z\right) = \frac{G'''}{D_{T}}\frac{z^{2}}{2} + C_{1} z + C_{2}$ (5).

# Imposing the two BCs yields the values of the constants C_1_ and C_2_, which may be substituted in Equation 5 to give:

# $C_{O2,T}\left(z\right) = C_{T} - \frac{1}{2} \frac{G'''}{D_{T}} \delta_{T}^{2} \left[1 - \left(\frac{z}{\delta_{T}}\right)^{2}\right] + \frac{G'''}{D_{T}} \delta_{T} \delta_{i,U} \left[1 - \frac{z}{\delta_{T}}\right]$ (5a).

# Equation 5a may be re-written in dimensionless form with the introduction of the Thiele modulus, ϕ, as follows:

# $\frac{C_{O2,T}\left(z/\delta_{T}\right)}{C_{O2,B}} = \frac{C_{T}}{C_{O2,B}} - \frac{1}{2} \varphi^{2} \left[1 - \left(\frac{z}{\delta_{T}}\right)^{2}\right] + \varphi^{2} \frac{\delta_{i,U}}{\delta_{T}} \left[1 - \frac{z}{\delta_{T}}\right]$ (5b).

# An expression for C_T_ and δ_i,U_ may be obtained by recalling that at z= δ_i,U_ C_O2,T_=0, and that at z=δ_T_ oxygen is supplied to tissue from the medium above. Imposing the first condition makes Equation 5a degenerate in a second degree equation in (δ_i,U_/ δ_T_) the solution to which gives:

# $\frac{C_{T}}{C_{O2,B}} = \frac{1}{2} \varphi^{2} \left(1 - \frac{\delta_{i,U}}{\delta_{T}}\right)^{2}$ (6).

# The second condition may be expressed in terms of a macroscopic mass balance on oxygen between the medium volume above the strip and the strip volume that gives:

# $k_{c} \left(C_{O2,B} - C_{T}\right) = A \left(\delta_{T} - \delta_{i,U}\right) G'''$ (7).

# Equation 7 may be rearranged in dimensionless form by introducing the mass Biot number Bi_m_ defined in Equation 2 to give:

# $\frac{C_{T}}{C_{O2,B}} = 1 - \frac{\varphi^{2}}{Bi_{m}} \left(1 - \frac{\delta_{i,U}}{\delta_{T}}\right)$ (7a).

# Equations 6 and 7a may be solved in a set to give the expression of δ_i,U_/δt reported in Equation 2 and of C_T_/C_O2,B_, as follows:

# $\frac{C_{T}}{C_{O2,B}} = \frac{1}{2} \varphi^{2} \left[1 - 2 \frac{\delta_{i,U}}{\delta_{T}} + \left(\frac{\delta_{i,U}}{\delta_{T}}\right)^{2}\right]$ (8).

# Substitution of Equation 8 for C_T_/C_O2,B_ in Equation 5b yields Equation 1.
