# Supplementary material for: Is oxygen availability a limiting factor for in vitro folliculogenesis?
Source: PLoS One. 2018 Feb 9;13(2):e0192501. doi: 10.1371/journal.pone.0192501 (PMC5806880; doi:10.1371/journal.pone.0192501)
Supplement: S2 Appendix — Details on how the concentration profile in ovarian tissue was obtained when ovarian tissue is cultured in gas-permeable dishes. (DOCX) [file pone.0192501.s002.docx]

# S2 Appendix

# When a strip of ovarian tissue is cultured in dishes with gas-permeable bottom (PD), assuming that an anoxic zone forms amid the strip thickness uncouples the mass balance equations for the transport of dissolved oxygen from the upper and the bottom surfaces. Under such conditions, the dissolved oxygen concentration profile in the uppermost part for the strip is well described by Equation 1. The dissolved oxygen concentration profile in the bottom part of the strip may be obtained from a steady-state mass balance on oxygen written about the control volume A dz (of infinitesimal thickness dz and surface area A equal to the strip area) in tissue near the bottom strip surface shown in Fig 1, and well described by Equation 4. In PD, Equation 4 is subject to the following boundary conditions:

# BC1 z=0 C_O2,T_=C_O2,TSB_=C_O2,B_ (9a)

# BC2 z=δ_i,B_ dC_O2,T_/dz=0 (9b).

# Imposing the two new BCs yields values of the constants C_1_ and C_2_ in Equation 5 that yield:

# $\frac{C_{O2,T}\left(z/\delta_{T}\right)}{C_{O2,B}} = \left[1 + \frac{1}{2} \varphi^{2} \left(\frac{z}{\delta_{T}}\right)^{2} - \varphi^{2} \frac{\delta_{i,B}}{\delta_{T}} \frac{z}{\delta_{T}}\right]$ (10).

# An expression for δ_i,B_ may be obtained by recalling that at z=δ_i,B_ C_O2,T_=0. Imposing such condition yields a value $\frac{\delta_{i,B}}{\delta_{T}} = \frac{\sqrt{2} }{\varphi}$that substituded in Equation 10 gives Equation 3.

# 
