# Supplementary material for: Is oxygen availability a limiting factor for in vitro folliculogenesis?
Source: PLoS One. 2018 Feb 9;13(2):e0192501. doi: 10.1371/journal.pone.0192501 (PMC5806880; doi:10.1371/journal.pone.0192501)
Supplement: S1 Table — Percentages of bovine follicle grading, staging and viability. H = Histology; V = Viability. Number of follicles analysed are indicated in brackets. (DOCX) [file pone.0192501.s003.docx]

|  | **GRADING** | | | **STAGING** | | | **VIABILITY** |
| --- | --- | --- | --- | --- | --- | --- | --- |
|  | I | II | III | PRIMORDIAL | PRIMARY | SECONDARY |  |
| D0  (H, 307; V, 254) | 27,5  (n=84) | 37  (n=114) | 35,5  (n=109) | 78  (n=240) | 19  (n=58) | 3  (n=9) | 92,5  (n=235) |
| D3 CDHV  (H, 242; V, 278) | 41  (n=99) | 31,5  (n=76) | 27,5  (n=67) | 16,8  (n=41) | 82,8  (n=200) | 0,4  (n=1) | 44  (n=122) |
| D3 PDHV  (H, 265; V, 312) | 35  (n=93) | 43,5  (n=115) | 21,5  (n=57) | 1,6  (n=4) | 98  (n=260) | 0,4  (n=1) | 81  (n=253) |
| D6 CDHV  (H, 287; V, 323) | 10  (n=29) | 48,5  (n=139) | 41,5  (n=119) | 2,6  (n=7) | 79,4  (n=228) | 18  (n=52) | 53  (n=171) |
| D6 PDHV  (H, 223; V, 236) | 35  (n=78) | 47  (n=105) | 18  (n=40) | 0,5  (n=1) | 79,5  (n=177) | 20  (n=45) | 78  (n=184) |
| D9 CDHV  (H, 275; V, 202) | 7  (n=19) | 42  (n=116) | 51  (n=140) | 1,6  (n=4) | 74,2  (n=204) | 24,2  (n=67) | 54  (n=109) |
| D9 PDHV  (H, 238; V, 364) | 40  (n=95) | 36,5  (n=87) | 23,5  (n=56) | 3,3  (n=8) | 71,2  (n=169) | 25,5  (n=61) | 76,3  (n=278) |

**S1 Table.** Experiment I: Percentages of bovine follicle grading, staging and viability. H=Histology; V=Viability. Number of follicles analysed are indicated in brackets.
