# Supplementary material for: Is oxygen availability a limiting factor for in vitro folliculogenesis?
Source: PLoS One. 2018 Feb 9;13(2):e0192501. doi: 10.1371/journal.pone.0192501 (PMC5806880; doi:10.1371/journal.pone.0192501)
Supplement: S2 Table — Percentages of bovine follicle grading, staging and viability. H = Histology; V = Viability. Number of follicles analysed are indicated in brackets. (DOCX) [file pone.0192501.s004.docx]

|  | **GRADING** | | | **STAGING** | | | **VIABILITY** |
| --- | --- | --- | --- | --- | --- | --- | --- |
|  | I | II | III | PRIMORDIAL | PRIMARY | SECONDARY |  |
| D0  (H, 273; V, 237) | 37  (n=101) | 33  (n=90) | 30  (n=82) | 85,5  (n=233) | 12  (n=33) | 2,5  (n=7) | 92,5  (n=219) |
| D6 PDHV  (H, 213; V, 173) | 60  (n=128) | 36,5  (n=78) | 3,5  (n=7) | 1,7  (n=4) | 78,8  (n=168) | 19,5  (n=41) | 75  (n=130) |
| D6 PDLV  (H, 287; V, 187) | 18  (n=52) | 53  (n=152) | 29  (n=83) | 2,5  (n=7) | 81,5  (n=234) | 16  (n=46) | 50  (n=94) |
| D6 CDHV  (H, 199; V, 148) | 11  (n=22) | 63  (n=125) | 26  (n=52) | 2,7  (n=6) | 79  (n=157) | 18,3  (n=36) | 60  (n=89) |
| D6 CDLV  (H, 271; V, 226) | 30  (n=81) | 46  (n=125) | 24  (n=65) | 3,2  (n=9) | 83  (n=225) | 13,8  (n=37) | 68  (n=154) |

**S2 Table.** Experiment II: Percentages of bovine follicle grading, staging and viability. H=Histology; V=Viability. Number of follicles analysed are indicated in brackets.
