# Supplementary material for: Is oxygen availability a limiting factor for in vitro folliculogenesis?
Source: PLoS One. 2018 Feb 9;13(2):e0192501. doi: 10.1371/journal.pone.0192501 (PMC5806880; doi:10.1371/journal.pone.0192501)
Supplement: S3 Table — Percentages of bovine follicle grading, staging and viability. H = Histology; V = Viability. Number of follicles analysed are indicated in brackets. (DOCX) [file pone.0192501.s005.docx]

|  | **GRADING** | | | **STAGING** | | | **VIABILITY** |
| --- | --- | --- | --- | --- | --- | --- | --- |
|  | I | II | III | PRIMORDIAL | PRIMARY | SECONDARY |  |
| D0  (H, 222; V, 143) | 30  (n=67) | 39  (n=86) | 31  (n=69) | 74  (n=164) | 23  (n=51) | 3  (n=7) | 93,4 |
| D6 PDHV AIR  (H, 187; V, 96) | 39  (n=73) | 27  (n=50) | 34  (n=64) | 19,3  (n=36) | 55,3  (n=103) | 25,4  (n=48) | 71,5  (n=69) |
| D6 PDHV 5%  (H, 148; V, 109) | 1  (n=1) | 1  (n=1) | 98  (n=146) | 11,4  (n=17) | 81,9  (n=121) | 6,7  (n=10) | 25,3  (n=28) |
| D6 CDHV  AIR  (H, 265; V, 121) | 29  (n=77) | 15  (n=40) | 56  (n=148) | 3,6  (n=10) | 71,4  (n=189) | 25  (n=66) | 48,5  (n=59) |
| D6 CDHV  5%  (H, 156; V, 118) | 3  (n=5) | 7  (n=11) | 90  (n=140) | 11,8  (n=18) | 80,7  (n=126) | 7,5  (n=12) | 18,6  (n=219) |

**S3 Table.** Experiment III: Percentages of bovine follicle grading, staging and viability. H=Histology; V=Viability. Number of follicles analysed are indicated in brackets.
