# Supplementary material for: Is oxygen availability a limiting factor for in vitro folliculogenesis?
Source: PLoS One. 2018 Feb 9;13(2):e0192501. doi: 10.1371/journal.pone.0192501 (PMC5806880; doi:10.1371/journal.pone.0192501)
Supplement: S4 Table — Percentages of human follicle grading, staging and viability. H = Histology; V = Viability. Number of follicles analysed are indicated in brackets. (DOCX) [file pone.0192501.s006.docx]

|  | **GRADING** | | | **STAGING** | | | **VIABILITY** |
| --- | --- | --- | --- | --- | --- | --- | --- |
|  | I | II | III | PRIMORDIAL | PRIMARY | SECONDARY |  |
| D0  (H, 273; V, 237) | 50,4  (n=138) | 28  (n=76) | 21,5  (n=59) | 77,9  (n=212) | 19,3  (n=53) | 2,8  (n=8) | 93,6  (n=222) |
| D6 CDHV  (H, 213; V, 173) | 12,3  (n=26) | 36,6  (n=78) | 51,1  (n=109) | 20,8  (n=44) | 75,5  (n=161) | 3,6  (n=8) | 47  (n=81) |
| D6 PDHV  (H, 287; V, 187) | 41,7  (n=120) | 21,7  (n=62) | 36,5  (n=105) | 25,5  (n=73) | 54,9  (n=158) | 19,5  (n=56) | 72,4  (n=135) |
| D9 CDHV  (H, 199; V, 148) | 19  (n=38) | 29  (n=58) | 52  (n=103) | 15  (n=30) | 80,5  (n=160) | 4,5  (n=9) | 39,2  (n=58) |
| D9 PDHV  (H, 271; V, 226) | 42,9  (n=117) | 25,2  (n=68) | 31,8  (n=86) | 20,6  (n=56) | 60,1  (n=163) | 19,2  (n=52) | 64,8  (n=146) |

**S4 Table.** Experiment IV: Percentages of human follicle grading, staging and viability. H=Histology; V=Viability. Number of follicles analysed are indicated in brackets.
